# Book Reviews

**Published:** 1894-10

**Authors:** 


### *BOOK REVIEWS.*

---

An International System of Electro-Therapeutics: for Students, General Practitioners, and Specialists. By Horatio R. Bigelow, M.D., and Thirty-eight Associate Editors. Thoroughly illustrated. In one large Royal Octavo volume, 1160 pages. Extra Cloth, $6.00 net; Sheep, $7.00 net; Half-Russia, $7.50 net. Philadelphia: The F. A. Davis Co., Publishers, 1914 and 1916 Cherry Street.

The author of this work has been assisted by some thirty-eight associates, which certainly makes the work a comprehensive one, covering the field in most all its phases. Yet we fear it lacks that simplicity which is so essential to a medical student, that he may fully understand and easily comprehend the many subjects of which it treats.

For the general practitioner it contains a fund of information well up to date in the latest methods and developments in electro-therapeutics, and even the specialists may glean many points of interest from its pages.

It contains an article on the necessity for Special Education in Electro-Therapeutics, and one on Electro-Physics, while under different headings and by various authors all varieties of electricity—batteries, currents, etc., are discussed, including Electro-Physiology and Electro-Diagnosis.

The various diseases and surgical conditions in which electricity has been found beneficial have been considered, the greatest space (276 pages) being devoted to gynecological and obstetrical subjects.

The various articles being written by different authors, there is some repetition of thoughts, while in some articles the pathology of disease is dealt with at too great length for a work of this character.

To the general practitioner who has some knowledge of the principles of electricity and is interested in electro-therapeutics, this work is to be recommended. In it he will find much of profit in the principles of electricity, as well as its actions physiologically and in pathological conditions, including its surgical use. R. R. K.

---

A TREATISE ON THE PRINCIPLES AND PRACTICE OF MEDICINE; DESIGNED FOR THE USE OF PRACTITIONERS AND STUDENTS OF MEDICINE. By Austin Flint, M.D., LL.D., Late Professor of the Principles and Practice of Medicine and Clinical Medicine in the Bellevue Hospital Medical College, New York, etc. Seventh edition, thoroughly revised by Frederick P. Henry A.M., M.D., Professor of the Principles and Practice of Medicine in the Woman's Medical College of Pennsylvania; Physician to the Philadelphia Hospital. Lea Brothers & Co., Philadelphia, Pa.

We are very glad to see this excellent work again brought before the public. It has been thoroughly revised and some valuable additions made, while the chapter on General Pathology has been omitted. This, we think, is best, for there are so many works on General Pathology, that it is not wise to give it space in a book on the practice of medicine.

This book is the best work we have seen on the special pathology of diseases. It gives the details of the special pathology of all the diseases discussed. There are many new articles in this edition not found in the older ones. Among these are articles on Pulsating Pleurisy, Weil's Disease, Beri-beri, Hereditary Chorea, Glanders, etc.

The chapter on Functional Diseases of the Stomach has been largely rewritten. We are glad to see the chapter on Dyspepsia omitted, as the term is largely used to cover ignorance on the part of the practitioner.

Taken all around this is one of the best works on the practice of medicine before the profession. J. L. C.

